# Long-term relapse: markers, mechanisms, and implications for disease management in alcohol use disorder

**DOI:** 10.3389/fpubh.2025.1706192

**Published:** 2026-01-07

**Authors:** John F. Kelly, Morgan Klein, Katherine Zeng, Sydney Manske, Alexandra Abry

**Affiliations:** Department of Psychiatry, Recovery Research Institute, Massachusetts General Hospital, Harvard Medical School, Boston, MA, United States

**Keywords:** addiction, disease management, long-term relapse, recurrence, relapse

## Abstract

**Objective:**

Much has been theorized and documented about factors involved in alcohol use disorder (AUD) relapse during the early months following a recovery attempt where biobehavioral classical conditioning (“cues/triggers”) and neurophysiological explanatory theories predominate. Little has been documented, however, about long-term relapse (LTR) factors following sustained AUD remission where self-regulation and stress and coping theories may predominate because LTR precursors are centered less around neurophysiological dysregulation and cue reactivity and more around factors such as lowered recovery vigilance, avoidant coping, or changes in recovery-support services (RSS) usage. Greater knowledge of factors involved in LTR could sensitize and empower clinicians to deliver more effective disease management protocols to monitor and intervene upon such risks *prior* to AUD recurrence.

**Methods:**

Cross-sectional, retrospective study of individuals in recovery from primary AUD (*N* = 50; 44% Female; 50% White) who had experienced LTR within the past 5 years following at least 1 year of remission (*M* years remitted prior to relapse = 3.6; range = 1–23) and assessed for any change in bio-psycho-social domains or RSS usage during the year prior to LTR, along with their attributions of factors' contribution to relapse (risk “potency”; i.e., didn't contribute, possibly, probably, or definitely, contributed). Research questions focused on the year preceding the LTR assessing: (1) prevalence and nature of the bio-psycho-social and RSS use changes and degree of attributed LTR risk potency; (2) number and type of definitely-contributing relapse factors within participants; (3) dynamic temporal onset and nature of high-risk LTR precipitants; (4) single most influential LTR risk factor.

**Results:**

Several bio-psycho-social and RSS changes occurred during the year preceding LTR varying in prevalence and potency. Some were prevalent, but not potent, in terms of definitely contributing to LTR (e.g., sleep, energy); others occurred infrequently, but were potent (e.g., physical pain, recreational drug use); others were both highly prevalent and highly potent (e.g., change in recovery vigilance). Within participants, median number of definitely contributing LTR factors = 4, covering 2 different domains, on average. Temporal accumulation of LTR risks tended to intensify toward the relapse horizon over the preceding year. The single most important relapse factor tended to cluster in psychological (e.g., recovery vigilance, mental health) and social domains.

**Conclusions:**

Findings have implications for long-term disease management during AUD recovery providing a set of potential preliminary markers and mechanisms that might be assessed, monitored, and, when necessary, intervened upon prior to the onset of heavy symptomatic alcohol use to prevent AUD recurrence.

## 1 Introduction

During the past 50 years of alcohol use disorder (AUD) research, much has been theorized and documented empirically regarding the factors involved in relapse in the early weeks and months following a recovery attempt (1–3). Because of the importance of initial metabolic and psychosocial stabilization in individuals' lives, much of AUD care has centered around withdrawal management and the operant and classically conditioned cues or triggers (e.g., certain people and places, as well as time of day/day of the week) that can precipitate use/harmful use of the drug. As such, the goal of improving the odds of achieving initial remission through treatment efforts has been focused on metabolic stabilization and on building the repertoire of cognitive and behavioral relapse prevention coping skills, and facilitating cognitive restructuring and de-conditioning of classically conditioned cues [e.g., (4)]. Most pharmacological trials and manualized psychosocial treatments have been developed for, and tested during, the early months of recovery stabilization [e.g., typically the first 12 weeks post-detoxification (2)]. A great deal of research conducted therefore has been focused on understanding the short-term biobehavioral vulnerabilities that can precipitate relapse as a means to better address them by developing, testing, and implementing critically important clinical protocols (2).

While vitally important, however, almost nothing is known about the factors involved in long-term relapse (LTR) after the achievement of full sustained remission (i.e., after one or more years of abstinence/non-symptomatic use), despite the fact that we have learned that stable recovery—wherein the risk of meeting criteria for an alcohol or other drug disorder in the following year is approximately the same as that of the general population - occurs only after about 5 years of continuous remission (5–8). Indeed, well-articulated and empirically-supported theories regarding factors involved in relapse risk during the *early months* of recovery exist, but these theories may have less applicability to relapse following full sustained remission. This is because, conceivably, neurophysiological dysregulation and post-acute withdrawal phenomena, and classically conditioned cue reactivity, are comparatively less salient following full sustained remission because it may have been years since the last use of alcohol, and central nervous system dysregulation could have re-adapted to abstinence and deconditioning occurred.

It is possible, however, that for some (e.g., those with more severe and chronic AUD histories), neurological, neurocognitive, and psychophysiological deficits (e.g., sleep, appetite, energy levels) could continue to confer long-term relapse risk even after a year or more of sustained remission. Such risks might also be exacerbated from a neuro-psychopharmacological perspective (e.g., through low level alcohol use, cannabis use, opioid medication exposure, tobacco use changes), kindling craving or interfering with neurological healing and recalibration, disrupting mood and emotion regulation. Indeed, prior research has found that among individuals in sustained AUD remission, those who continue to use alcohol even at lower levels are much more susceptible to subsequent AUD recurrence (2, 9, 10). Yet, it is more likely that, during these later stabilized recovery stages, other self-regulation (11, 12) and stress and coping theory (13, 14) related psychosocial processes may be more significant. Specifically, decay in cognitive vigilance and recovery focus (e.g., keeping AUD recovery as a daily priority) or resolve; shifts in attitudes toward the need for, or use of, recovery support services (e.g., AA, SMART Recovery; counseling); social network changes (e.g., acquiring new heavy drinking friends/colleagues as a result of job promotion); inability to manage acute or chronic negative (distress) or positive (eustress) life-course events, such as changes in environment (e.g., buying a new house in a new area); work, education, and income changes (new job/job promotion, retirement; returning to college), social role transitions (e.g., birth of a child, children leaving home, deaths); or coping with medical illness onset or offset (e.g., heart disease, cancer). Thus, such risks relating to LTR, might be categorized broadly into bio-psycho-social, as well as recovery support services (RSS), change domains.

Given there are currently no clinically robust observable AUD relapse risk biomarkers (e.g., blood or urinalysis) that can alert clinicians to potential impending relapse *prior* to the onset of symptomatic heavy drinking when someone is still in sustained AUD remission, such delineation of risk must still rely on clinical interview and patients' self-report. For practitioners engaged in long-term disease management for AUD (e.g., AUD recovery monitoring in primary care settings), it is critical, therefore, to have knowledge of the nature and potency (i.e., relapse risk potential) of the variety of possible bio-psycho-social and RSS use changes that are shown to be associated with subsequent LTR in order to assess for them and alert AUD patients currently in remission who may be unwittingly on a trajectory that is placing them at higher risk for LTR, so that they can “course correct” before it's too late, prior to resumption of heavy symptomatic alcohol use. Yet, whereas sporadic and anecdotal accounts of the factors precipitating LTR are sometimes suggested (e.g., the patient stopped attending recovery support services such as AA), more precise, comprehensive, empirical documentation of the nature, aggregation, and risk potency of, and dynamic temporal changes involved in, such risk factors preceding LTR, is sorely lacking.

To this end, this study was designed to begin to systematically document the nature, prevalence, potency, and dynamic temporal onset of the factors precipitating LTR through addressing several fundamental clinically relevant research questions: (1) What are the commonly reported bio-psycho-social and RSS use changes that occur in the year prior to a relapse following full sustained remission for individuals in long-term recovery from AUD; and, to what degree of certainty do such individuals attribute such changes as contributing to their relapse. (2) What is the prevalence and nature of the reported “definitely” contributing relapse factors within persons. (3) When do such potential high-risk warning signs occur and intensify during the year prior to LTR, and (4) What is the single most influential reported contributor of LTR relapse among individuals in prior full sustained AUD remission.

## 2 Methods

### 2.1 Participants

Participants (*N* = 50) were recruited from December 2023 until November 2024. Advertisements were placed on Craigslist, Facebook, Instagram, and the Mass General Brigham online platform Rally. Individuals interested in participating were contacted by study staff via phone or email and completed a phone screening with trained study staff to determine eligibility. To be eligible for participation, individuals needed to: (1) be age 18 or older, (2) meet the Diagnostic and Statistical Manual of Mental Disorders, Fifth Edition [DSM 5 (15)] criteria for lifetime history of AUD, (3) be abstinent from alcohol for the past 90 days or be in early remission from AUD (participant does not meet more than one DSM 5 criterion for AUD), (4) have had a recurrence of AUD symptoms within the last 5 years, before which they must have been in remission for at least 1 year, and (5) listed alcohol as the primary substance from which they were in recovery.

Of the 840 people who were interested in the study, 358 were screened for study eligibility via phone. Of these who were screened, 220 were ineligible, an additional 41 were excluded due to being either duplicates of other screens or for providing fraudulent answers; 97 met study inclusion criteria. After providing verbal consent through a fact sheet, study participants were provided a link to complete the self-administered, online surveys and were scheduled for an approximately 1-h Zoom interview with study staff. One eligible individual did not complete the consent process and thus, 96 people were enrolled in the study. Six individuals missed their appointment and could not be rescheduled, and 17 individuals were withdrawn after internal data validity checks. Of the 73 completed interviews, 23 were removed from the final dataset due to validity concerns. The final sample (*N* = 50) represents 14% of the 358 screened for eligibility.

### 2.2 Procedures

Interested individuals who contacted the study team by email, phone, or Rally were asked to participate in an approximately 15-min phone screen to assess their eligibility. Study data were collected and managed using REDCap electronic data capture tools hosted by Mass General Brigham Research Computing, Enterprise Research Infrastructure and Services (ERIS) group (16).

Following consent and prior to the assessment interview, participants completed a brief set of self-administered surveys via REDCap. Surveys consisted of questions regarding demographics, as well as lifetime substance use, addiction treatment, mental health diagnosis and treatment, and recovery support service utilization. Staff reviewed survey responses for completeness and validity prior to the interview.

To be eligible, during the screening process, individuals underwent a validated semi-structured interview to capture AUD status at various important timepoints. To help minimize retrospective recall bias, the following timeline of events had to have occurred within the past 5 years. First, participants had to have met criteria for AUD in the year prior to achieving at least 1 year of full sustained remission, and, then, subsequently, report a relapse to alcohol use (typically involving a return to frequent heavy use, related consequences, and that lasted for more than 1 week and up to many months or longer). Also, at the time of the study interview, participants also had to be at least in early remission from AUD (i.e., no AUD symptoms in the past 90 days) and either be abstinent or report no more than National Institute on Alcohol Abuse and Alcoholism (NIAAA)-defined low-risk alcohol use and report no days of other frequent, heavy, other drug use that interfered with functioning during the past 3 months. To state this again in more common language, at some point during the past 5 years, participants in sustained AUD remission must have relapsed, and now, be sober/in early remission again for a minimum of 90 days at the time of the interview without any recent heavy alcohol or other drug use exposure.

Interviews were conducted over Zoom and lasted roughly 1 h and were audio recorded for transcription purposes. Interviews delved into life factors that may or may not have contributed to a recurrence of AUD symptoms. These factors covered four domains: biological (e.g., sleep, nutrition, pain, mental and physical health, substance use), psychological (e.g., quality of life, recovery capital, recovery vigilance), social (e.g., life events, social network), and health services (e.g., medication, therapy, mutual-help organization [MHO] participation). The participant was prompted to expand on each theme. When the theme was fully explored and no further change factors/relapse precipitants were reported, the interviewer shifted the focus to the next theme. Interviewers attempted to establish a general timeline—when each theme emerged, if it did, during the 12-month period leading up to the reported AUD relapse.

Interviews were transcribed verbatim using a transcription service (TranscribeMe) and then reviewed by research staff to ensure accuracy. Following review, recordings were deleted to ensure confidentiality. Subjects received $50 for participation in our study, in the form of a gift card (Amazon, Target, Dunkin').

For the purposes of current study, we summarize and report here the types of reported relapse precursors across the four domains (biological, psychological, social, health services) including each participants' confidence in the degree to which each reported factor they believed contributed to their relapse (i.e., did not contribute, possibly contributed, probably contributed, definitely contributed), along with the timing (month of onset) of each factor's occurrence in the year prior the relapse.

The study protocol and procedures were approved by the Mass General Brigham Institutional Review Board. This mixed quantitative-qualitative study was not pre-registered, thus findings should be considered exploratory.

### 2.3 Measures

#### 2.3.1 Screening measures

To assess whether participants successfully met the critical AUD milestones (see procedure above), the AUD Diagnostic Assessment Research Tool [DART (17)]. Additionally, if participants reported yes to having used any alcohol at all since they got back into recovery following their relapse, recent heavy alcohol exposure was assessed according to the NIAAA criteria with women asked: “In the past 90 days, have you had 3 or more drinks on any single day or consumed more than 7 drinks in any given week.” And men were asked, “In the past 90 days, have you had 4 or more drinks on any single day or consumed more than 14 drinks in any given week.”

To additionally rule out potential participants reporting frequent recent use of other drugs besides alcohol, during screening, participants answered a series of questions about 15 substances/classes of substances from the Global Appraisal of Individual Needs [GAIN-I (18)] (e.g., alcohol, marijuana, cocaine, heroin, amphetamine/methamphetamine, etc.). If participants endorsed using any of the 15 substances 10 or more times in their lifetime, they were asked: (1) In the past 3 months (90 days), on how many days did your use of drugs interfere with your functioning [FORM-90 (19)]. If 4 or more days of use was reported, participants were excluded.

#### 2.3.2 Demographics

Participants reported their date of birth, sex assigned at birth, gender identity, race, ethnicity, where they were living in the past 3 months, current marital status, sexual orientation, education, parental/guardian education, current employment status, and total annual household income using standardized validated items from the GAIN-I (18).

#### 2.3.3 Substance use

The study surveys asked participants a series of questions about 15 substances/classes of substances (hereafter simply referred to as substances) from the GAIN-I (18) (e.g., alcohol, marijuana, cocaine, heroin, narcotics other than heroin (e.g., pharmaceutical opioids), etc.) (see above under Screening Measures).

#### 2.3.4 Mental health

Participants were asked if they had ever been told that they had a mental health condition by a doctor, nurse, or counselor. If, yes, they were asked which mental health condition among any one of 22 substance use/mental health conditions (e.g., Agoraphobia, Anorexia nervosa, Bipolar disorder (I or II), Bulimia nervosa, Delusional disorder, Dysthymic disorder, Generalized anxiety disorder, Major depressive disorder etc.) or “other” mental health diagnosis not listed, which if selected, were prompted to specify [GAIN-I (18)].

Medication prescribed by a physician or medical practitioner for a mental health condition (lifetime use; not including treatment for substance use or health problems) was assessed by asking which medication they had ever been prescribed (1) antidepressants, (2) anti-anxiety medication, (3) anti-psychotics, (4) mood stabilizers, (5) stimulants, (6) painkillers, (7) medications for sleep 1, (8) medications for sleep 2, and other psychiatric medications) [GAIN-I (18)].

#### 2.3.5 Treatment for alcohol and drug use problems

The Form-90 [FORM-90 (19)] was used to assess prior treatments received in their lifetime including emergency room visits, hospital admissions, and prescribed anti-craving/anti-relapse medications (e.g., acamprosate, naltrexone/depot naltrexone, disulfiram, nalmefene, topiramate, baclofen).

#### 2.3.6 12-step/MHO attendance history

Participation at any one of 11 different MHOs (e.g., AA, NA, MA, CMA, CA, SMART Recovery, LifeRing, Moderation Management, Celebrate Recovery, Women for Sobriety etc.), with an “other” option specified by participant was used, was assessed using the Multidimensional Measure of Mutual-Help Assessment Scale [MMHAS (20)]. For each MHO, participants, participants reported whether they ever attended regularly (at least once per week).

#### 2.3.7 Recovery support services and formal treatment programs

History of participation in nine formal psychosocial treatment and recovery support services was assessed using the GAIN-I (18): (1) Outpatient addiction treatment; (2) Inpatient or residential treatment; (3) Alcohol or drug medical detoxification services; (4) sober living environment (e.g., halfway house, Oxford house, sober dorm); (5) recovery high school; (6) collegiate recovery program/community; (7) recovery community center (RCC)/Peer Recovery Community Center/Recovery Café; (8) faith-based recovery services (e.g., addiction recovery support group provided by a church, synagogue, mosque); or (9) state or local recovery community organization (RCO).

#### 2.3.8 Online resources and social network sites

The questionnaire assessed participants' history of using various online/remote recovery resources. Potential online and mobile technologies included (1) online MHO meetings; (2) Discussion forums for alcohol/drug recovery; (3) social network sites (e.g., Facebook, YouTube, Instagram), but focused on your recovery/change attempt; (4) Recovery-focused social network sites (e.g., In The Rooms); (5) Text message programs for alcohol/drug recovery; (6) Smartphone apps for alcohol/drug recovery; (7) Live chats (e.g., with a recovery coach, therapist, peer); (8) Online educational resources (e.g., Recovery Answers website, NIAAA Alcohol Effects and Help Page; (9) Other (to be specified by the participant).

#### 2.3.9 Study interview guide

At the start of the interview, participants were asked to confirm the date they initiated their recovery. Once the recovery date was confirmed, participants were asked: (1) Why did you initiate recovery in *[date stated]* and (2) What was helping you maintain your recovery? Participants were then asked to confirm their relapse date. Once confirmed, participants were asked: what was going on in the year leading up to your relapse? When needed, study staff would prompt the participant by asking about (1) significant events and (2) vacations, holidays, and personal memories that stand out during the 1 year leading up to their relapse [similar to the Timeline Follow-Back Procedure (21)]. Participants were also asked: (1) Is there anything that changed in your attitude toward your recovery/emotional state and (2) Was there anything you were doing differently? Study staff prompted participants with questions about the year leading up to their relapse for approximately 30 min.

Participants were asked questions across bio-psycho-social and recovery support services use areas. The questionnaire was devised by the first author (JK) for the purposes of this study. Participants were asked whether there were changes in any of the following specific areas:

Participants were asked nine biological/health questions: (1) Were you having problems with sleep? (2) Were you feeling less or more energetic than usual? (3) Were you having problems with appetite or eating habits (e.g., eating too little or too much)? (4) Were you experiencing significant weight gain or loss? (5) Were you suffering from chronic/ongoing pain? (6) Were you using recreational drugs to get high or change how you were feeling? (7) Were you starting to use tobacco, or quitting tobacco? (8) Were you having physical health problems? and (9) Was there a change in your medications?

Participants were asked seven social questions: (1) Were you spending more time around alcohol? (2) Were you becoming socially isolated? (3) Were you feeling lonely? (4) Did you experience a change in your employment? (5) Did you lose someone close to you? (6) Was there a change in your living situation? (7) Was there a change in your financial situation?

Participants were asked six psychological questions: (1) Were you feeling more impulsive than usual (i.e. acting without considering the consequences, or thinking things through? (2) Did you begin to focus less on your own recovery? (3) Were you feeling less confident that you could sustain your recovery? (4) Were you satisfied with life? (5) Did engagement in compulsive behaviors, such as gambling, gaming, sex, exercise, shopping increase? (6) Were you having trouble with mental health symptoms (such as depression or anxiety)?

Participants were asked four health services questions: (1) Was there a change in the SUD treatment you were getting? (2) Was there a change in the way you were attending meetings with MHO/12-step organizations (such as SMART Recovery and Alcoholics Anonymous? (3) Was there a change in your use of recovery support services (such as recovery coaching, recovery community centers, or recovery housing)? (4) Was there a change in your use of psychological medications or counseling?

Additionally, participants were asked the following open-ended questions: (1) Are there any other factors that occurred in the 12 months prior to your relapse that I haven't asked you about, and (2) Did any of these questions jog your memory about that year?

Relapse Attribution Definiteness: For each change element that participants' endorsed, they were asked to approximate the date the change began, and to rate how that factor contributed to their relapse (“To what degree did you feel that this factor contributed to your relapse?) rated on a four-point scale (did not contribute = 0, possibly contributed = 1, probably contributed = 2, definitely contributed = 3).

Major Reason for Relapse: At the end of the interview, participants were asked: “As you reflect on your relapse, what do you think was the major reason why you relapsed”. Responses were categorized by the research team into one of the same four broad category domains: biological/health, psychological, social, and RSS.

### 2.4 Analytic strategy

#### 2.4.1 Quantitative analysis

**Figure 1:**
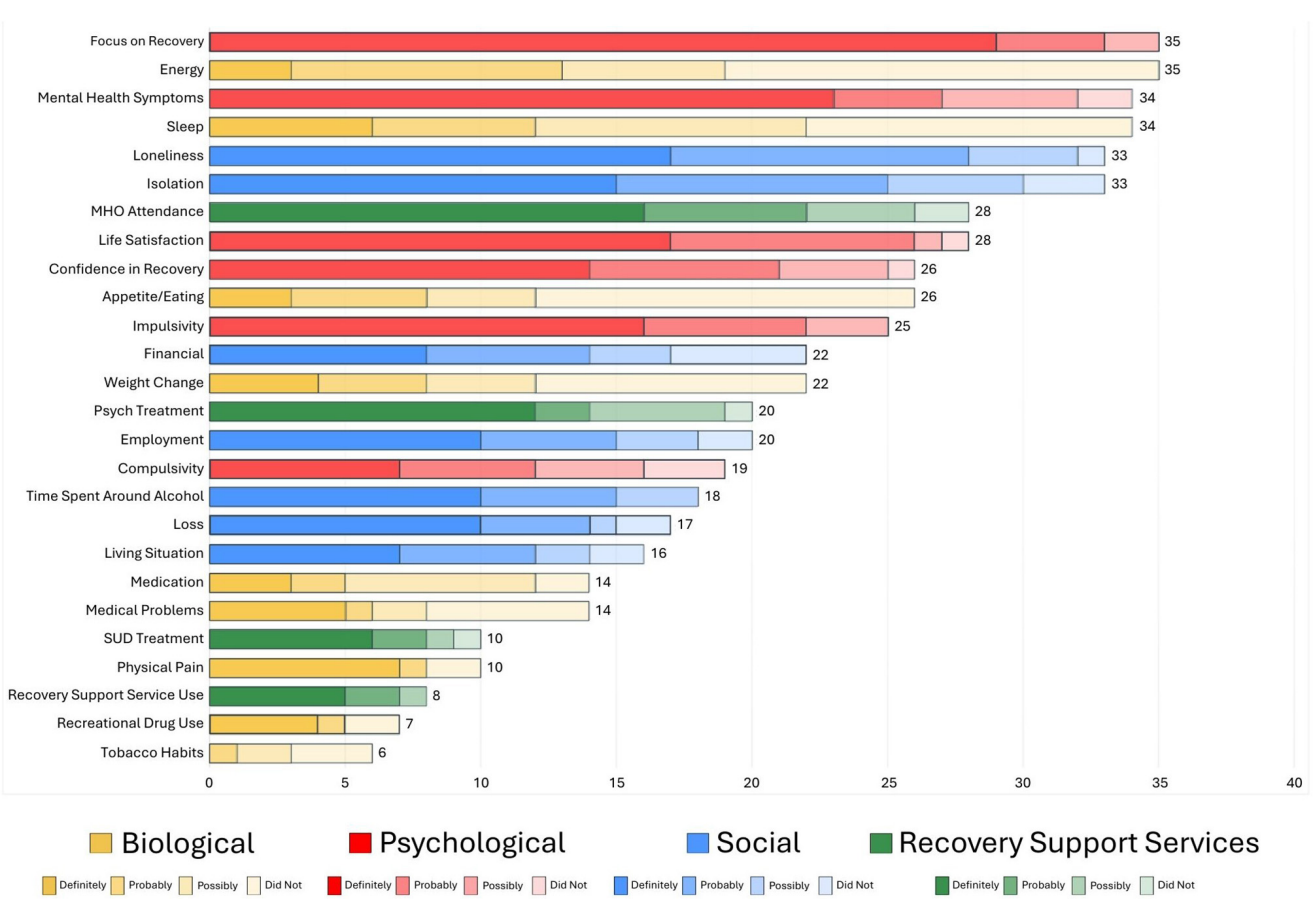
Ranking by frequency of factors reported as changing in the year prior to long-term relapse.

**Figure 2:**
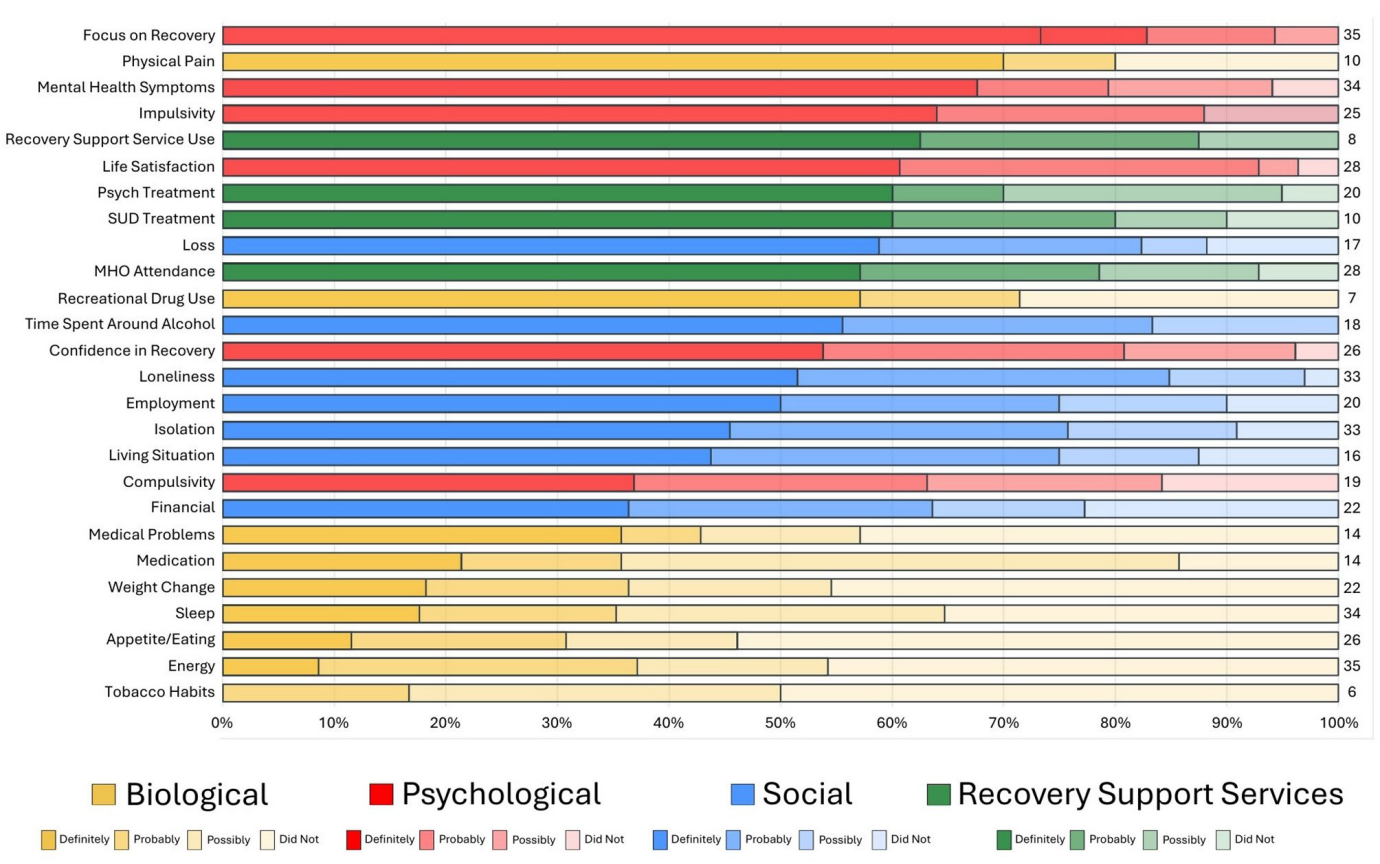
Ranking by potency of factors precipitating long-term relapse.

Descriptive summary statistics were used to describe the characteristics of the sample including means, standard deviations, frequencies/percentages, and ranges (Table 1). For the relapse precipitants interview questionnaire, endorsed categories and items were organized and summarized graphically in a color-coded fashion (by biological, psychological, social, RSS usage domains) in terms of how frequently each type of domain/sub-domain was reported across the sample along with the degree of attributional definiteness in causing or precipitating their relapse (i.e., did not contribute, possibly contributed, probably contributed, definitely contributed; Figures 1, 2). Data were also summarized by color-coded domain in terms of when they were estimated to have begun during the year prior to the relapse event (Figure 6).

**Table 1:** Participant characteristics and service utilization (*N* = 50).

**Variable**	**Mean/%**	**(SD/*n*)**
Age	39.91	(9.85)
**Gender identity**		
Man	56	(28)
Woman	44	(22)
**Sex**		
Male	52	(26)
Female	46	(23)
Hispanic or Latino (% yes)	8	(4)
**Race**		
American Indian or Alaskan Native	2	(1)
Asian	4	(2)
Black or African American	44	(22)
White or Caucasian	50	(25)
Other	4	(2)
**Sexual orientation**		
Heterosexual	80	(40)
Homosexual	10	(5)
Bisexual	2	(1)
Pansexual	6	(3)
**Income (i.e., total household past year)**		
Less than $10,000	8	(4)
$10,000-$49,999	44	(22)
$50,000 or more	48	(24)
**Current employment**		
Full-time	48	(24)
Part-time	30	(15)
Unemployed	18	(9)
**Marital Status**		
Single	46	(23)
In a relationship	50	(25)
Previously married	10	(5)
**Living Situation (i.e., past 90 days)**		
With family	48	(24)
With friend(s) or non-family	8	(4)
Alone in own dwelling	38	(19)
Other	6	(3)
**Education**		
High school or less	8	(4)
Some college or other degree	38	(19)
BA or higher	52	(26)
High school or less	8	(4)
Some college or other degree	24	(12)
BA or higher	60	(30)
**Mental health**		
**Lifetime diagnosis (% yes)**		
Any	62	(31)
Mood disorder	52	(26)
Anxiety disorder	46	(23)
Substance use disorder	78	(39)
Post-Traumatic Stress Disorder (PTSD)	44	(22)
Other disorder	38	(19)
Psychiatric medication use (% ever)	56	(28)
**Utilization of addiction and recovery services**		
**Formal treatment (% ever)**		
Outpatient addiction treatment	56	(28)
Alcohol/drug detoxification	44	(22)
Inpatient or residential treatment	38	(19)
Emergency room treatment	44	(22)
Hospital admission (≥1 night)	38	(19)
Medication for alcohol use	30	(15)
**Recovery support services (% ever)**		
Sober living environment	36	(18)
Education-based service	4	(2)
Recovery community center or organization (RCC/RCO)	26	(13)
Faith-based recovery services	20	(10)
**Mutual help (% ever)**		
Alcoholics anonymous	88	(44)
Other 12-step MHO	62	(31)
Other MHO	22	(11)
None	10	(5)
**Online/remote recovery resources (% ever)**		
Online meetings	50	(25)
Discussion forums for alcohol/drug recovery	28	(14)
Social media sites focused on recovery/change attempt	42	(21)
Recovery-focused social network sites	16	(8)
Text messaging programs for alcohol/drug recovery	8	(4)
Smartphone apps for alcohol/drug recovery	20	(10)
Live chats (e.g., with a recovery coach)	24	(12)
Online educational resources	18	(9)
**Substance use**		
Age at first alcohol use	16.08	(4.71)
Age at first regular alcohol use	19.67	(4.51)
Mean (SD) years in full sustained remission at time of relapse	3.59	(3.94)
Range	1–22.5	
Median	2.25	

**Figure 3:**
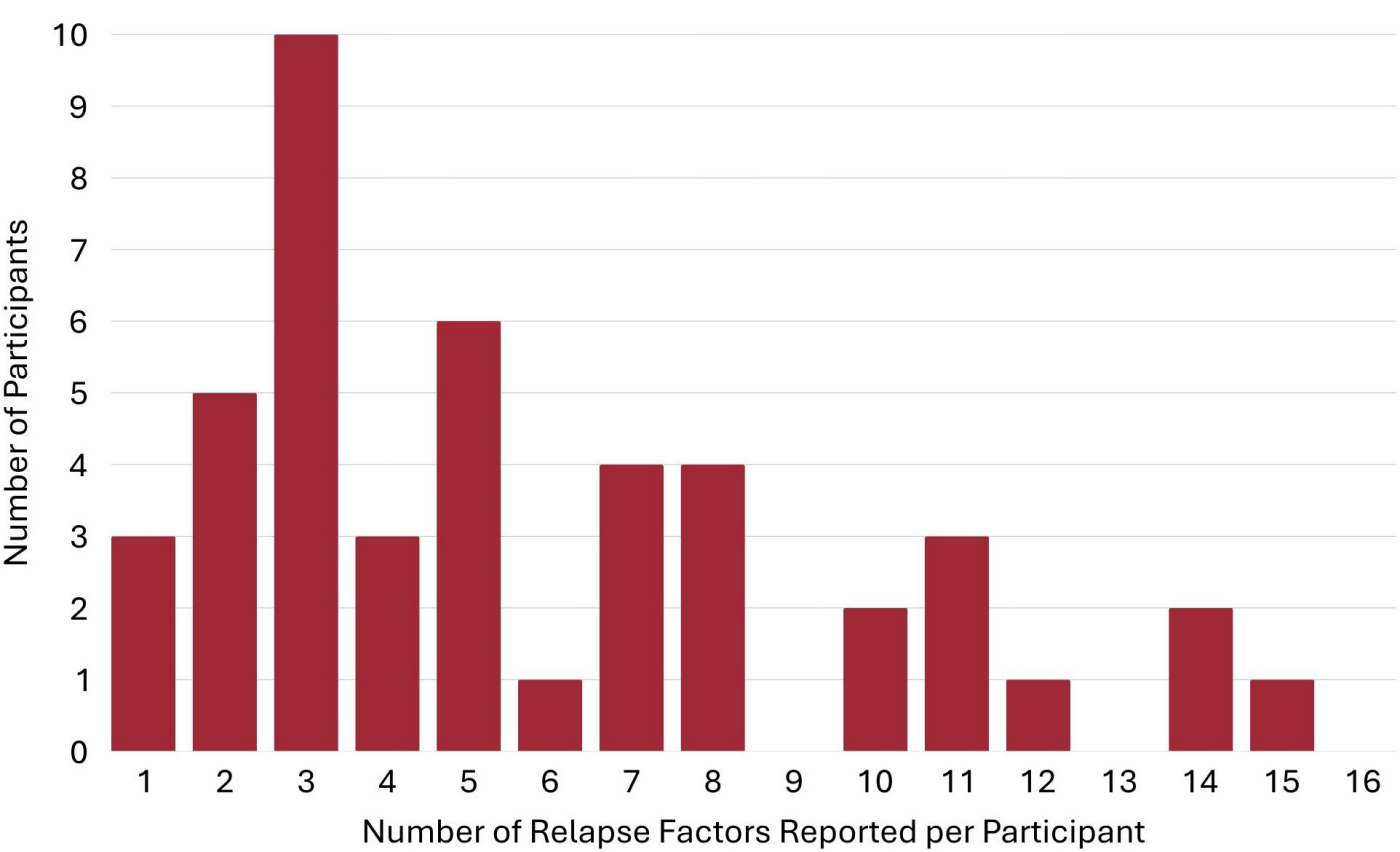
Number of factors endorsed by each participant that ‘'definitely contributed‘' to relapse.

**Figure 4:**
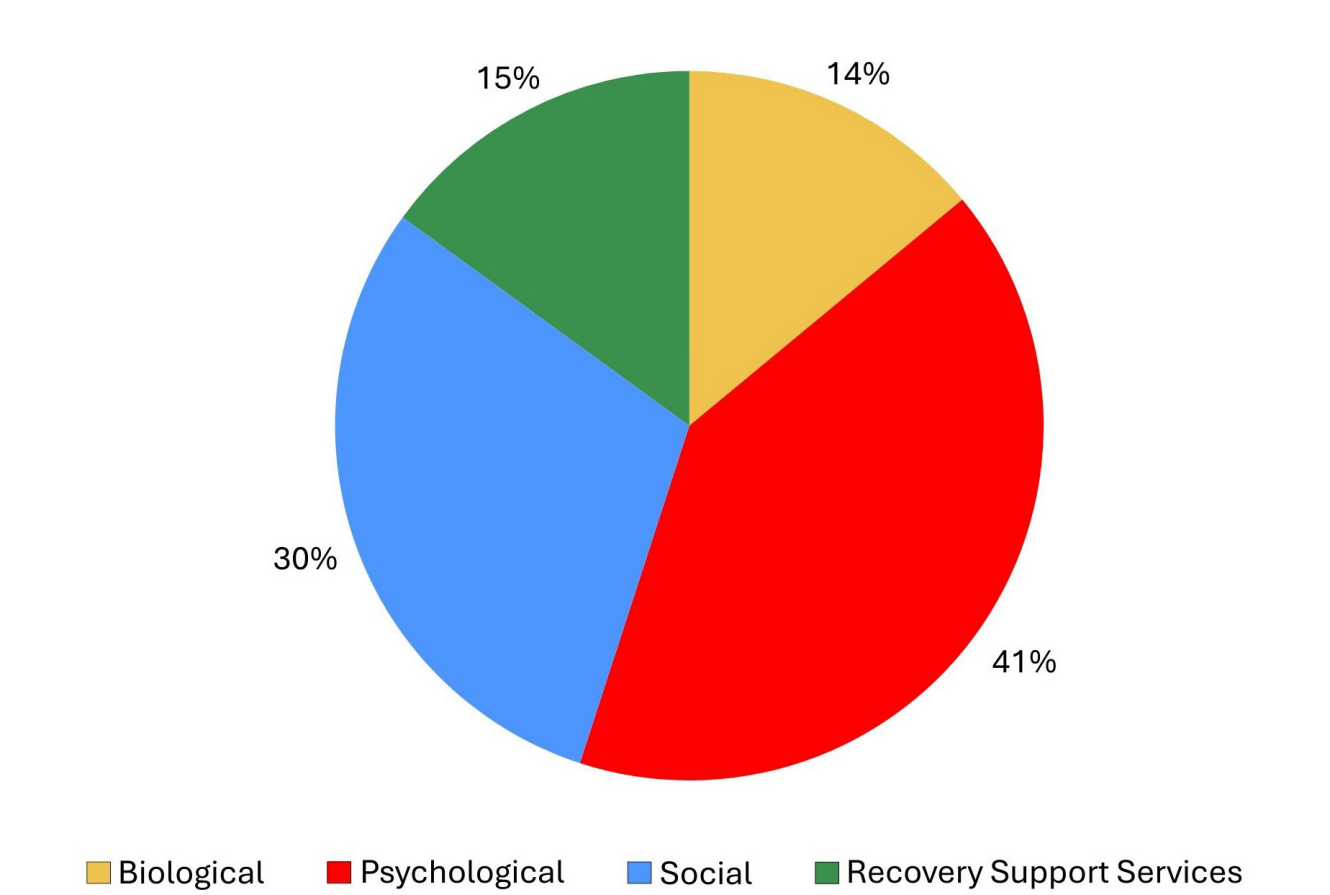
Proportion of different domain factors that ‘'definitely contribute‘' to long-term relapse.

**Figure 5:**
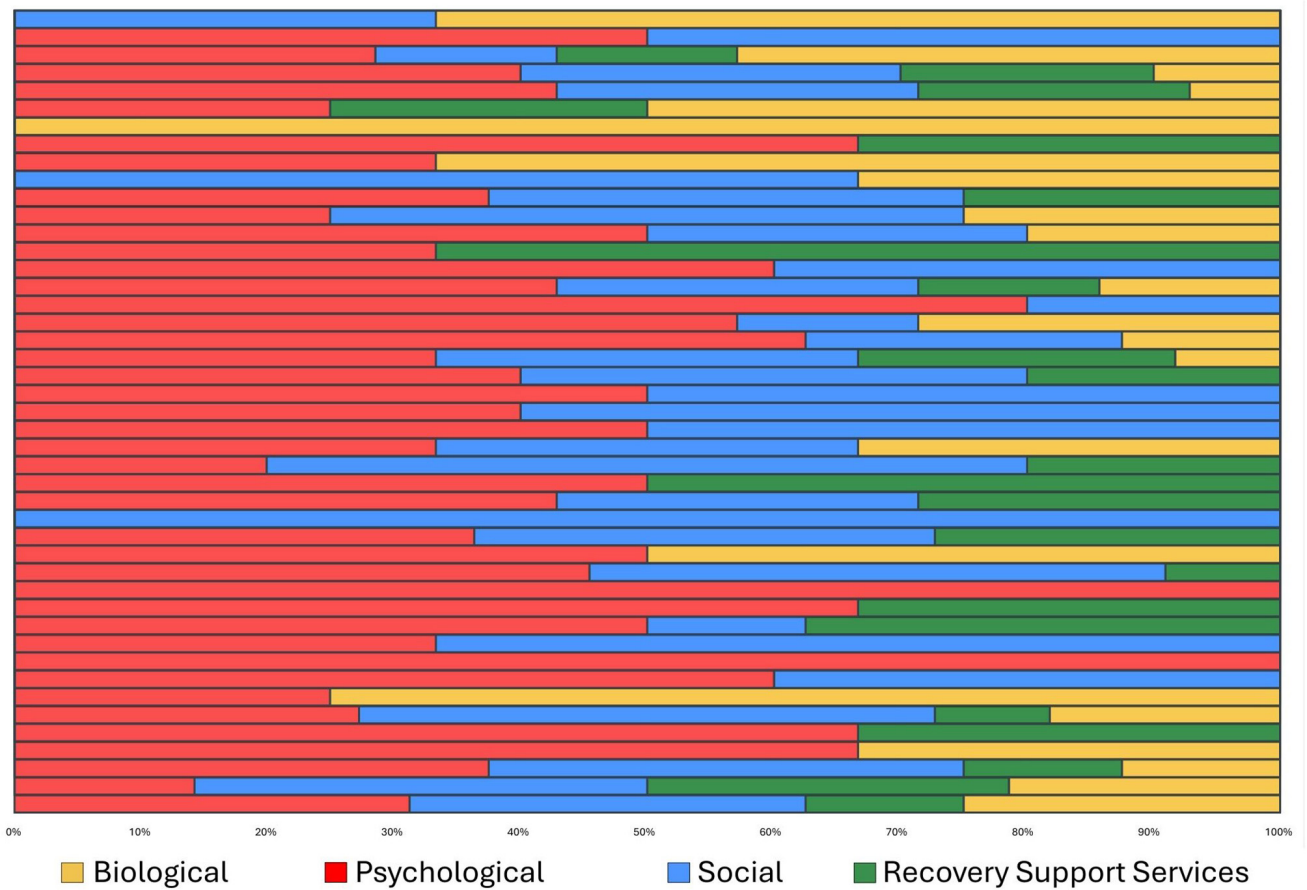
Domains of items that ‘'definitely contributed‘' to long-term relapse by participants.

**Figure 6:**
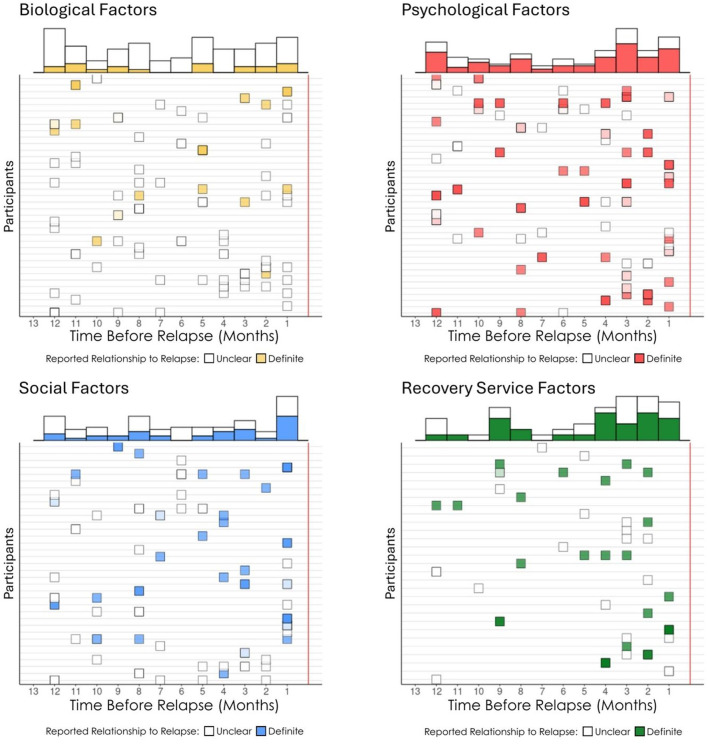
Temporal sequencing of factors that “definitely contribute” in the year prior to the relapse. Boxes represent factors reported to have changed in the year prior to relapse. Darker boxes result from an overlap of multiple changes indicated on the same time point.

**Figure 7:**
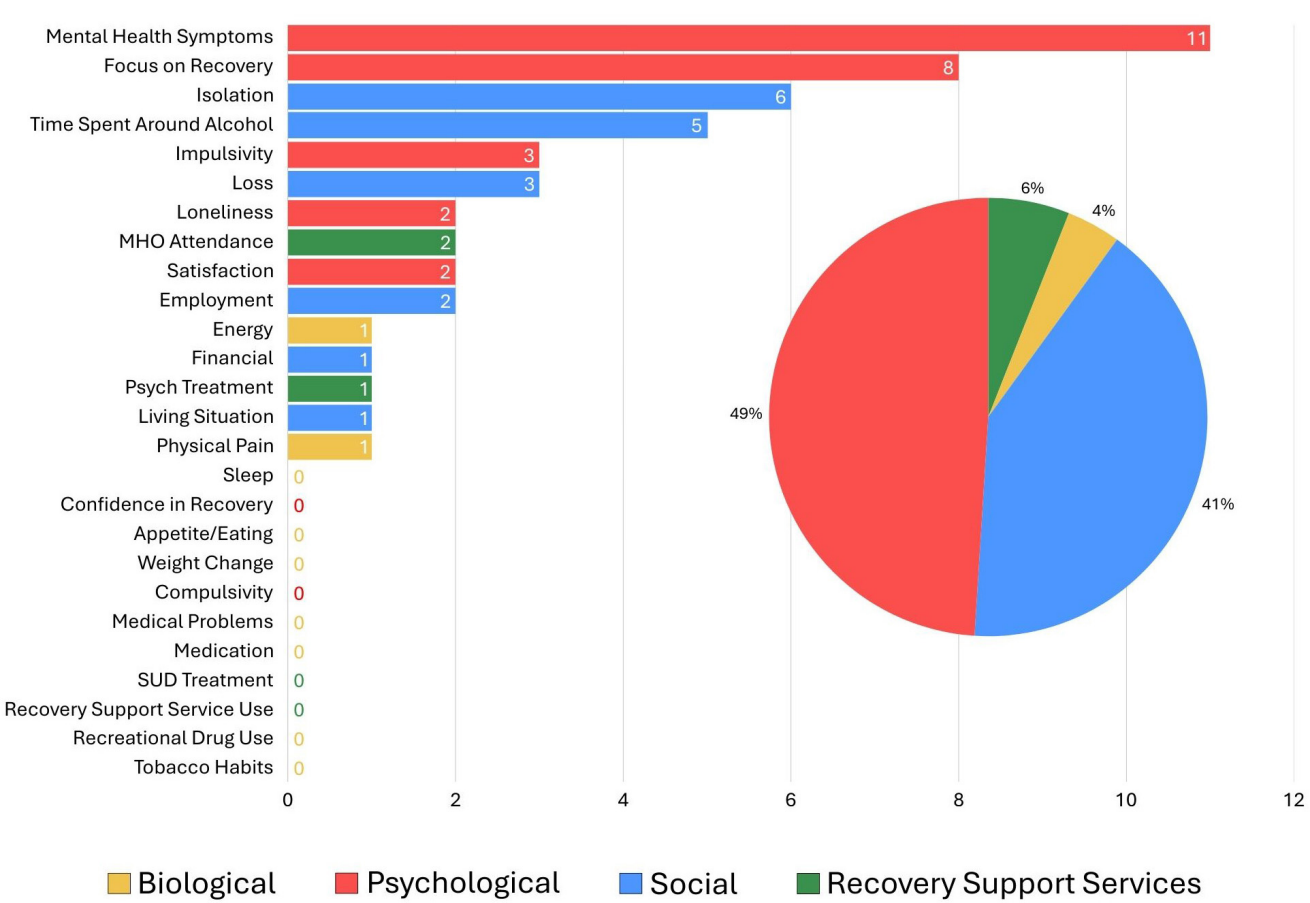
Self-reported most major reason for return to use.

Data were summarized and organized in keeping with the study's four central research questions:

- What are the commonly reported bio-psycho-social and RSS use changes that occur in the year prior to a relapse following full sustained remission for individuals recovering from AUD and to what degree of certainty do such individuals attribute such changes as contributing to their relapse. First, we examined and graphically presented which color-coded domains and related factors were reported as changing in the year prior to the relapse following at least one year of full sustained remission. Of the factors reported as changing during the year prior to the relapse, we examined the degree to which participants reported each of these as contributing to their relapse ranging from “did not contribute”, to “possibly contributed”, to “probably contributed”, to “definitely contributed”. These were then ranked by the individual-level factors that “definitely contributed” to their relapse as a proportion of the reported factors that changed during the year prior to relapse and color-coded by domain (Figures 1, 2).
- What is the frequency and nature of the reported contributing relapse factors. We examined the number of total “definitely contributing” factors that were reported as contributing to their relapse and examined and graphically presented the degree to which these fell into each of the four categories (biological, psychological, social, recovery support services; Figures 3–5).
- When do such potential high-risk warning signs occur during the year prior to a long-term relapse. We explored and graphically presented the temporal onset sequencing of definitely contributing relapse factors and summarized by domain (Figure 6).
- What is the single most influential reported contributor of relapse among individuals in prior full sustained remission who have subsequently relapsed. Responses to the open-ended question, “As you reflect on your relapse, what do you think was the major reason why you relapsed?” were coded using our framework with the 26 items covering the four domains: biological/health (9 items), social (7 items), psychological (6 items), and health services (4 items). Two members of the research team (SM, KZ) reviewed participant responses independently and coded each quotation using this framework. There was moderate interrater reliability (63.3% agreement; Cohen's kappa = 0.54). The majority of coding discrepancies were resolved by discussion with the two members (84.2%). The remaining three responses initially coded as “other” were reclassified following discussion with the larger team. One response was removed due to insufficient information, resulting in a final sample of *n* = 49. This list of the most influential reported relapse precipitants were summarized, rank ordered according to frequency, and graphically presented (Figure 7).

## 3 Results

### 3.1 Sample characteristics

As shown in Table 1, approximately half the sample was female, with an average age of about 40 years old. Most identified as of either Black or White racial background, and non-Hispanic. Most were employed either part- or full-time, and about half were currently in a relationship and about half reported living with family. Income was generally low to moderate and educational attainments was quite with high the majority reporting have some college participation or achieving a BA or higher. About two-thirds reported a lifetime mental health diagnosis with about half reporting prior use of psychotropic medications. In terms of prior addiction service usage, about one-third to just over half reported either medication, withdrawal management, or some type of outpatient or inpatient treatment. The vast majority reported prior use of AA and many reported use of other 12-step MHOs or other recovery support services with about one-third reporting a history of residing in a sober living environment. Digital recovery support services use were also quite commonly reported. Age of first alcohol use was around age 16 with regular (at least once per week) reported by age 19 years old.

### 3.2 Frequency of reported bio-psycho-social and recovery support services use changes occurring in the year prior to LTR following full sustained remission and degree of reported attributional certainty such changes contributing to the LTR

Figures 1, 2 illustrate both the frequency (far right-hand column) and “potency” (degree of ascribed certainty in causing the individuals' relapse represented by the different domain color shades) of each of the reported potential long-term relapse factors with Figure 1 rank ordered by frequency and Figure 2 by potency (% “definitely contributed”).

As shown, the top factor both in terms of frequency and potency was a change in individuals' Focus on Recovery (*N* = 35). Of note, when reported as changing during the year prior to the relapse, it was always considered to be at least “possibly contributing” and more than 80% of time as “definitely” having contributed. Two biological factors (energy level and sleep) were the second and fourth most common reported elements that changed in the year preceding the relapse, respectively, but both of these were only rarely reported as definitely contributing to relapse. The third most common change reported was in mental health challenges, which was also a potent relapse risk factor with close to 70% rating this change as definitely contributing to their relapse. Two social factors, loneliness and social isolation, were ranked fifth and sixth in terms of most commonly reported elements that changed during the year preceding relapse, and were also quite potent, with about half of participants reporting such changes as definitely contributed to their relapse. Mutual-help organization participation changes were the seventh most common and also quite potent, with close to 60% reporting the change as definitely contributing to their relapse. Three psychological factors—life satisfaction, confidence in recovery (recovery self-efficacy) and impulsivity, were the eighth, ninth, and eleventh most common reported elements that changed, with approximately 50–60% of participants rating these elements as definitely contributing to their relapse. Two other biological factors—appetite/eating, and weight change, were ranked the tenth and thirteenth most common change elements, but these were only rarely reported as definitely contributing to relapse.

In terms of social factors, changes in financial situation, employment, time spent around alcohol, loss, and living situation, were also quite commonly reported as changing in the year prior the relapse, and were also relatively high-risk with approximately 40–50% of participants reporting that these definitely contributed to relapse. Other biological factor changes such as medications, medical problems, physical pain, recreational drug use, and tobacco use changes were less common elements noted to have changed in the year preceding the relapse and were generally low potency with the exception of physical pain and recreational drug use, where approximately 70% and 58% of participants, respectively, reported them as definitely contributing to their relapse. Two health service usage factors were less frequently reported as changing, but when they did, they were generally of high-risk relapse potency: changes in recovery support services use and change in SUD treatment use, where approximately 65% in each domain reported such changes as definitely contributing to their relapse.

Of note in Figure 2, which is rank ordered from the most to least potent relapse risk factors in terms of definiteness, a glance at the color coding of the domains reveal clusters of biological and social factors in the lower half of the chart, indicating about 50% or less describing these as definitely contributing to their relapse. Thus, with the exception of physical pain which possessed high relapse risk potency, there was a general trend for social, and especially biological factors, to possess lower, or low, relapse risk potency compared to psychological and RSS use change domains, which tended to contain higher potency relapse contributing factors.

### 3.3 The frequency and nature of reported “definitely” contributing long-term relapse factors within participants

As shown in Figure 3, the number of definitely contributing factors ranged from 0 to 16 within participants, with a very skewed distribution. The median was 4 per person (*M* = 5.2, SD = 4.05) and this average of four definitely contributing factors, covered an average mean of 2.3 (SD = 1.17) different domains (biological, psychological, social, RSS). As highlighted in Figure 4, of the reported definitely contributing relapse factors, some individuals reported factors in a single domain (e.g., biological), but many reported factors covering all four domains as definitely contributing (Figure 4). The pie chart in Figure 5, shows the proportion of each domain items that were ascribed as definitely contributing to relapse.

### 3.4 Temporal onset and relapse risk potency of changes occurring during the year prior to long-term relapse

As shown in Figure 6, we constructed a timeline of the temporal sequencing of the onset of the reported change factors for each participant separated by domain (biological, psychological, social, services) along with their ascribed relationship to participants' relapse dichotomized as either unclear (not related, possibly related, probably related) or definite (definitely related). Marginal histograms summarized by month at the top of each domain figure have hollow bars reflecting the total number of change events reported with the proportion of “definitely” contributing factors filled in each respective domain color. As noted above, there was a trend for participants to ascribe greater confidence in contribution to relapse for psychological and treatment and recovery support service changes in the year prior the relapse than for the biological or social domains (see Figure 6). Of note also, was that there tended to be a general linear increase over time from left to right leading up to the relapse horizon, in both the number of change factors reported, as well as the proportion of definitely contributing factors, with the exception of biological factors which tended to show a more flat distribution.

### 3.5 Single most influential reported contributors of relapse

Summarized results from the final question that participants were asked, “What do you think is the major reason you relapsed?”, are presented in Figure 7, ranked from most to least frequent and color coded according to domain. As shown, the highest proportion of reported reasons were psychological (53% of participants) or social (37% of participants) in nature, with the psychological domain factors, mental health symptoms and focus on recovery, reported as the highest single most commonly referenced influential relapse risk factors overall, followed by the social domain factors, isolation and time spent around alcohol. Also, in the social domain, loss, employment, and living situation, were reported as major relapse reasons, but much less frequently. Similarly, within the psychological domain, impulsivity, loneliness, and life satisfaction, were reported as the major relapse causing changes, but these were infrequent. Major relapse reasons that fell into either the biological or RSS related realms, were represented by only 6% and 4% of participants, respectively. Two reported individual factors each fell into these two domains with MHO attendance and psychiatric treatment falling into the latter, and energy and physical pain falling into the former.

## 4 Discussion

While much is known about the theories and factors involved in short-term relapse as people try to stabilize and establish some initial early AUD remission, very little is known empirically about the factors involved in long-term relapse following a year or more of full sustained remission. Greater knowledge and conceptual understanding about the kinds of risks that individuals encounter prior to losing their sobriety, is of central importance, as AUD recurrence can be devastating and deadly. In terms of clinical models of long-term disease management in the treatment of clinically prevalent AUD, these so-called “remission-based warning signs” once detected might be highlighted, discussed, and addressed with the aid of a primary care, or other knowledgeable clinician, before they lead to resumption of problematic alcohol use and AUD recurrence. Given the lack of focus in this area to date, the current study is significant, innovative, and timely, in that it sheds some preliminary light on this important topic. Findings highlight a number of significant changes occurring in the year prior to a relapse following, often, years of full sustained AUD remission, many of which were attributed to causing AUD relapse with a high degree of certainty and, thus, may serve as useful set of preliminary markers or potential mechanisms that might be monitored in long-term disease management care protocols to help prevent AUD recurrence (e.g., see Appendix I).

Many types of biological, psychological, social, and addiction-related health services factors were noted by participants to have changed in the year preceding relapse. Such change factors varied in both frequency as well as relapse potency. Some were quite common, but not very potent—in terms of being attributed to definitely causing a relapse (e.g., energy levels, sleep changes)—whereas others were both common and potent (e.g., focus on recovery). Still others were comparatively infrequently reported, but potent or highly potent (e.g., recreational drug use, physical pain).

Several existing theories may be useful in helping to explain the occurrence of these factors and their relationship to long-term relapse explicated herein. As noted in the paper introduction above, theories based in neurophysiological post-acute withdrawal and cue reactivity, while highly applicable in models of early recovery stabilization (22), would appear to have less applicability in long-term relapse given the central nervous system healing and neurological and endocrinological recalibration that would have occurred in the years since last alcohol exposure (23). This lack of fit with such theories, might be said to be highlighted by the relatively less significant contribution of biological factors in reported relapse. More applicable with the relapse markers and mechanisms discovered here, are stress and coping (13, 14), self-regulation [e.g., 11], behavioral economic (24), and social identity (25), theories. Also, neurocognitive aging effects could be at play given the age-range and long-term remission durations (prior to relapse) of some of the cases (e.g., as long as 20+ years in sustained remission), which could affect aspects of memory, risk-appraisal, and decision-making (26).

More specifically, the top factor in terms of frequency of occurrence and potency in the year prior to the relapse was the change in focus on recovery—this aspect of continued cognitive vigilance is a central feature in self-regulation theory [e.g., 12] specifically in maintaining adequate healthy and functional self-regulation individuals must continually be aware of, and appraise, address, and successfully cope with, dynamic elements that might disrupt or distract the equilibrium. Further, underlying such a shift may be elements integral to social identity and behavioral economic theories of addiction recovery as the salience of being a “recovering person” may lose centrality in favor of more novel identities and competing rewards and reinforcers that, paradoxically, have emerged due to individuals' successful liberation from addiction and the accrual of recovery-related benefits [also referred to as “recovery capital” (27)] (32).

It is sometimes said that the word “slip” often used to describe a return to alcohol use following a period of cessation (e.g., “Jim had a slip”) among individuals with AUD attempting to achieve stable recovery, stands for “Sobriety Losing Its Priority”. What stands out in our set of findings here, is that a reduction in cognitive recovery vigilance may be a potent marker to continually assess for and address among individuals in long-term AUD remission. Phenomenologically, in the natural history of AUD recovery, as time in remission begins to accrue over many years, oftentimes homelife stabilizes and improves, relationships heal or new ones obtained, better employment and educational opportunities present themselves, and physical health and general resiliency all get better (24, 28). As alluded to above, this can lead naturally to positive distractions that may displace the focus on recovery-specific goals and activities in favor of new, and oftentimes positive, opportunities, that ironically have arisen because of individual's successful enduring recovery. This does not mean that such positives should not occur, but that such positive adaptations are part of recovery and need to be negotiated and successfully accommodated into the repertoire of activities within the recovery landscape while individuals continue to prioritize recovery and maintain cognitive recovery vigilance.

The popular “one day at a time” maxim of Alcoholics Anonymous, thus, may not only serve to assuage the angst caused by the potentially long-term depressogenic thought regarding, “how am I going to stay sober for the rest of my life”, but also may likely be borne of the kind of bitter experience of exactly the phenomenon herein where there is great potential for some individuals with long-term stable remission to take one's proverbial eye off the ball over time and focus elsewhere apparently at the peril of increasing relapse risk.

When one contextualizes this with the other findings here such as the fact that many people report several potent factors occurring simultaneously or close together during the year prior to their relapse and that are attributed as definitely contributing to relapse, it could also signify that the observed potent lack of recovery vigilance also may lead to a negative cascade in increased vulnerability that implicates other risk factors such as attenuation of treatment and recovery support services use (particularly reducing MHO participation)—now perceived to be unnecessary—resulting in a potential accelerating accumulation of risk that leads toward an implicit relapse horizon where resumption of alcohol use once again looks like a good idea—despite the enormous prior suffering that pushed the same individual into making often radical lifestyle changes to accommodate and facilitate the achievement of AUD remission to begin with. That said, the exact temporal causal sequencing of these multiple potent intersecting risks within persons remain to be clarified. It's equally plausible, for example, that the onset of mood and/or anxiety symptom changes might be more endogenous and biologically cause (e.g., during menopause in women) and subsequently interfere with a continued recovery priority focus. Further research is needed to untangle such intersecting risk elements. Of note, when asked to single out the most important contributor to relapse, most participants reported a psychological or social factor. Specifically, mental health symptom challenges and focus on recovery, impulsivity, and loneliness within the psychological domain; and isolation, time spent around alcohol, and loss within the social domain. Although more research is clearly needed in this regard, these types of changes detected as occurring under the auspices of AUD disease management protocols may require particular highlighting and proactive clinical intervention to help reduce subsequent relapse risk.

### 4.1 Limitations

There is clearly much to investigate in this area and there are many limitations inherent in the current study design and analyses, which need to be carefully considered when drawing conclusions and generalizations from the findings reported here. The sample size, while diverse in terms of sex and race, was quite small (*N* = 50) and mostly early middle-aged, and whereas study findings do offer important preliminary insights into this phenomenon, the sample could hardly be considered representative of the AUD long-term relapsing population. That said, inclusion criteria by necessity, contained several very specific boundaries (e.g., participants need to meet DSM 5 AUD criteria, then achieve full sustained remission for at least 1 year and then relapsed and now be at least in early remission all within the past 5 years) limiting the pool of eligible participants and speed of recruitment in the context of our fairly brief study timeline. Also, retrospective recall, analysis, and appraisal of past events, are all susceptible to cognitive biases involved in the processes of construction of memory and meaning-making, which could have affected reported factors and their attributed relapse risk potency estimation in unknown ways. That said, to address these, we did our best to limit the sample to having had to experience a long-term relapse within the past 5 years, and currently have returned to at least early remission status (i.e., no/subthreshold AUD symptoms within the past 90 days) and otherwise have no, or only very limited, alcohol or other drug exposure within the past 90 days to help minimize such bias and prevent recent heavy use potentially impairing current cognitive clarity. Also clear, was that the long-term relapse risk factors reported were many and several were potently related to increasing risk within persons. This finding underscores a multifaceted and dynamic long-term relapse risk scenario involving several, likely intersecting, factors that warrant continued untangling in terms of their temporal sequencing and nature. For example, as alluded to above, is it the onset of potentially endogenous mood and anxiety symptom changes that interfere with a continued recovery priority focus, or, are such potent mental health symptom challenges more exogenous, caused by a lack of recovery prioritization and relegation of recovery support service usage, resulting in decreased ability to manage the eustress and distress associated with navigating their recovery journey all within the broader context of the adaptations needed to adjust to, and address, broader developmental life-course challenges. Further research is needed to uncover and discover more about this. Furthermore, definitive bio-psycho-social categorization of the uncovered relapse markers/mechanisms herein are arguable (e.g., “focus on recovery” may be a psychological construct reflecting cognitive vigilance but also could be underwritten by more affective motivational importance). Finally, despite the limitations of this cross-sectional retrospective design, the phenomenon of long-term relapse is difficult to study even in prospective longitudinal research designs, due to likely “assessment reactivity” that could well raise consciousness and produce therapeutic cognitive and behavioral changes around the very phenomena under investigation (e.g., asking about focus on recovery or mental health symptoms or recovery support health services changes may well increase awareness among participants that could lead to adaptive changes ultimately preventing relapses that may have occurred without such questioning). Such therapeutic effects from assessment reactivity are known to occur and have begun to be clarified and their empirical magnitude documented [e.g., (29–31)]. As with all research questions, however, valid causal conclusions will be drawn ultimately from a mix of different research designs that all converge with consistency and coherence on a set of common findings.

### 4.2 Conclusions and clinical implications

In addition to the great need for more research into this important clinical and public health area, the array of potent biological, psychological, social, and recovery health services factor changes that occurred in the year preceding long-term AUD relapse detected in this study, highlight several markers and potential mechanisms that could be incorporated into a checklist (see Appendix I) for patients to complete in ongoing visits during long-term recovery monitoring. Such a list as those in Figures 1, 2, the presence of one or more of which, could alert a clinician to focused discussion and intervention or, otherwise, be used as a stimulus and foundation for broader clinical discussion about the need for continued AUD recovery focus and successfully facing and addressing recovery and life course challenges, ultimately may help prevent further morbidity and mortality that can arise from long-term AUD recurrence.

## Data Availability

The raw data supporting the conclusions of this article will be made available by the authors, without undue reservation.
