# Supplementary material for: Long-term relapse: markers, mechanisms, and implications for disease management in alcohol use disorder
Source: Front Public Health. 2026 Jan 7;13:1706192. doi: 10.3389/fpubh.2025.1706192 (PMC12819679; doi:10.3389/fpubh.2025.1706192)
Supplement: Supplementary file 1 [file Table_1.docx]

**APPENDIX I**

**POTENTIAL DISEASE MANAGEMENT AND MONITORING QUESTIONNAIRE FOR USE IN CLINCIAL PATIENT-ORIENTED RESEARCH ON ALCOHOL USE DISORDER RECOVERY AND LONG-TERM RELAPSE BASED ON STUDY FINDINGS**

---------------------------------------------------------------------------------------------------------------------------------------------------

**For Patients:**

**Rate your level of agreement with the following statements about significant changes you may have experienced since your last appointment**

**Biological**

|  | **Not at all** | **A little bit** | **Moderately** | **Quite a bit** | **A lot** |
| --- | --- | --- | --- | --- | --- |
|  | **0** | **1** | **2** | **3** | **4** |
| I have been having problems with sleep. |  |  |  |  |  |
| I have been experiencing changes in my energy levels. |  |  |  |  |  |
| I have been having problems with appetite and/or eating habits. |  |  |  |  |  |
| I have been experiencing significant weight changes. |  |  |  |  |  |
| I have been suffering from chronic/ ongoing pain. |  |  |  |  |  |
| I have been using recreational drugs to get high or change how I am feeling. |  |  |  |  |  |
| I have been making changes in my use of tobacco (started using or quitting tobacco). |  |  |  |  |  |
| I have been having physical health problems. |  |  |  |  |  |
| My medications have changed. |  |  |  |  |  |

**Psychological**

|  | **Not at all** | **A little bit** | **Moderately** | **Quite a bit** | **A lot** |
| --- | --- | --- | --- | --- | --- |
|  | **0** | **1** | **2** | **3** | **4** |
| I have been feeling more impulsive than usual |  |  |  |  |  |
| I have been focusing less on my own recovery |  |  |  |  |  |
| I have been feeling less confident I can sustain my recovery |  |  |  |  |  |
| I have been feeling dissatisfied with my life |  |  |  |  |  |
| I have been feeling more compulsive than usual |  |  |  |  |  |
| I have been having trouble with mental health symptoms |  |  |  |  |  |

**Social**

|  | **Not at all** | **A little bit** | **Moderately** | **Quite a bit** | **A lot** |
| --- | --- | --- | --- | --- | --- |
|  | **0** | **1** | **2** | **3** | **4** |
| I have been spending more time around alcohol. |  |  |  |  |  |
| I have become more socially isolated. |  |  |  |  |  |
| I have been feeling lonely. |  |  |  |  |  |
| My employment situation has changed. |  |  |  |  |  |
| I have been affected by the loss of someone close to me. |  |  |  |  |  |
| There has been a change in my living situation. |  |  |  |  |  |
| There has been a change in my financial situation. |  |  |  |  |  |

**Recovery Support Services**

|  | **Not at all** | **A little bit** | **Moderately** | **Quite a bit** | **A lot** |
| --- | --- | --- | --- | --- | --- |
|  | **0** | **1** | **2** | **3** | **4** |
| There has been a change in the addiction treatment I have been getting. |  |  |  |  |  |
| There has been a change in the way I attend my mutual-help/12-step meetings. |  |  |  |  |  |
| There has been a change in my use of recovery support services (e.g., recovery coaching, peer recovery support centers) |  |  |  |  |  |
| There has been a change in my use of psychological medications or counseling. |  |  |  |  |  |

**Example Scoring**

**Biological Score: _____/36**

**Psychological Score: ____/24**

**Social Score: ____/28**

**Recovery Support Service Score: ____/16**

**Total score: ___/ 104**

**NOTE:** **Any items** that patients endorse as having occurred could be raised as a point of clinical discussion.
